# Dynamic Correlations between Intrinsic Connectivity and Extrinsic Connectivity of the Auditory Cortex in Humans

**DOI:** 10.3389/fnhum.2017.00407

**Published:** 2017-08-11

**Authors:** Zhuang Cui, Qian Wang, Yayue Gao, Jing Wang, Mengyang Wang, Pengfei Teng, Yuguang Guan, Jian Zhou, Tianfu Li, Guoming Luan, Liang Li

**Affiliations:** ^1^Beijing Key Laboratory of Epilepsy, Epilepsy Center, Department of Functional Neurosurgery, Sanbo Brain Hospital, Capital Medical University Beijing, China; ^2^Beijing Hospital Beijing, China; ^3^School of Psychological and Cognitive Sciences and Beijing Key Laboratory of Behavior and Mental Health, Key Laboratory of Machine Perception (Ministry of Education), Peking University Beijing, China; ^4^Beijing Institute for Brain Disorders Beijing, China

**Keywords:** auditory evoked potential, auditory cortex, granger causal analysis, stereo-electroencephalography, intrinsic/extrinsic connectivity

## Abstract

The arrival of sound signals in the auditory cortex (AC) triggers both local and inter-regional signal propagations over time up to hundreds of milliseconds and builds up both intrinsic functional connectivity (iFC) and extrinsic functional connectivity (eFC) of the AC. However, interactions between iFC and eFC are largely unknown. Using intracranial stereo-electroencephalographic recordings in people with drug-refractory epilepsy, this study mainly investigated the temporal dynamic of the relationships between iFC and eFC of the AC. The results showed that a Gaussian wideband-noise burst markedly elicited potentials in both the AC and numerous higher-order cortical regions outside the AC (non-auditory cortices). Granger causality analyses revealed that in the earlier time window, iFC of the AC was positively correlated with both eFC from the AC to the inferior temporal gyrus and that to the inferior parietal lobule. While in later periods, the iFC of the AC was positively correlated with eFC from the precentral gyrus to the AC and that from the insula to the AC. In conclusion, dual-directional interactions occur between iFC and eFC of the AC at different time windows following the sound stimulation and may form the foundation underlying various central auditory processes, including auditory sensory memory, object formation, integrations between sensory, perceptional, attentional, motor, emotional, and executive processes.

## Introduction

In humans, passive listening to sound stimuli activates both the auditory cortex (AC) and some cortical regions that do not belong to the typical auditory system (Ackermann et al., 2001; Brown et al., 2004; Londei et al., 2007). Listening to either speech or music sounds not only initializes the bottom-up and top-down signal propagations between the early-stage AC and the association AC (Fontolan et al., 2014; Potes et al., 2014) and those between the association AC and the inferior frontal cortex (Potes et al., 2014), but also activates both the superior and inferior regions of ventral motor cortex (Cheung et al., 2016) and the posterior part of the superior temporal gyrus (STG) and the precentral gyrus (PreG) (Potes et al., 2012). These reports suggest that the AC both sends bottom-up signals to and receives top-down signals from higher-order cortical regions that underlie various auditory processes, such as speech and music perception. Also, cortical auditory activity can be enhanced by both top-down endogenous interpretation (Iversen et al., 2009) and attentional processing (Sussman et al., 2002; Debener et al., 2003), leading to task-specific response plasticity of the AC (Polley et al., 2006). Thus, investigation of the temporal dynamics of signal propagations between the AC and its connected cortical regions (even under passive listening conditions) is critical for understanding the cortical mechanism underlying auditory processing.

Intracranial electroencephalographic (EEG) recordings have been used for investigating neural mechanisms underlying auditory processing with both a high spatial resolution and a high temporal resolution (Lachaux et al., 2007; Edwards et al., 2009; Sinai et al., 2009; Korzeniewska et al., 2011; Mesgarani and Chang, 2012; Pasley et al., 2012; Mesgarani et al., 2014; Nourski and Howard, 2015). Importantly, intracranial EEG recordings can be used for clarifying the direction of signal propagations across brain regions. However, studies using intracranial EEG recordings in humans to examine the temporal dynamic of signal propagations between the AC and its connected cortical regions have rarely been reported.

Although both the bottom-up and top-down processes can be triggered automatically by a sound and play a role in exchanging information between the AC and non-auditory regions (O’Connor, 2012; Sohoglu et al., 2012), it is not clear how extrinsic functional connectivity (eFC) between the AC and higher-order non-AC cortices is associated with intrinsic functional connectivity (iFC) within the AC.

One of the core questions about brain networks is how different networks cooperate during perceptual/cognitive processing (Bressler and Menon, 2010). If investigation of interactions between the inter-regional bottom-up eFC and top-down eFC is critical for understanding the mechanisms underlying perceptual/cognitive processing (Pardo et al., 1991; Corbetta et al., 2000; Alain et al., 2001; Serences et al., 2005; Bonte et al., 2006; Buschman and Miller, 2007; Budinger et al., 2008; Chiu and Yantis, 2009; Elhilali et al., 2009; Salmi et al., 2009; Chica et al., 2011; Fontolan et al., 2014), it is even more critical for understanding these mechanisms to investigate the relationship between iFC and eFC of sensory cortices (Ghazanfar and Schroeder, 2006; Buschman and Miller, 2007; Bressler and Menon, 2010; Fontolan et al., 2014).

Bidirectional interactions have been discovered between the primary AC and the associate AC (Fontolan et al., 2014). iFC of the AC is important for spectro-temporal analyses, feature extraction/integration, and learning-induced reorganization (Griffiths and Warren, 2002; Eggermont, 2007; Gourévitch and Eggermont, 2010). It is of interest to know whether iFC of the AC both bottom-up modulates higher-order non-auditory cortices and is top-down modulated by the high-order cortices.

In this study, using simultaneously recorded, multiple intracranial stereo-electroencephalographic (sEEG) recordings, we examined the dynamic pattern of correlation between iFC of the AC and either bottom-up eFC from the AC to higher-order cortices or top-down eFC from higher-order cortices toward the AC. The directionality and strength of iFC and eFC were examined by Granger causality (GC) analyses using broadband electrophysiological signals. The test-retest reliability were also examined for further clinical applications. The hypothesis of this study is that iFC within the AC may be associated with not only bottom-up eFC from the AC to some higher-order non-auditory cortices but also top-down eFC from certain higher-order non-auditory cortices to the AC. More in detail, in the earlier time windows after the sound onset, iFC of the AC may be more associated with out-going eFC with higher-order non-auditory cortices, but in the later time windows, iFC of the AC may be more associated with in-coming top-down eFC from higher-order auditory cortices.

## Materials and Methods

### Participants

Ten people suffering from drug-refractory (pharmaco-resistant) epilepsy (3 females and 7 males, aged from 15 to 28 years old; *mean* = 22.7 years, *SD* = 4.1 years, see in **Table 1**), who were recruited in the Sanbo Hospital of Capital Medical University, participated in this study. The participants were undergoing long-term invasive sEEG monitoring to identify their seizure foci. All the participants had normal pure-tone hearing thresholds between 0.125 and 4 kHz (confirmed by tuning-fork tests, Tschiassny, 1946) and provided informed consent for their participation. The experimental procedures were approved by the Ethics Committee of the Sanbo Hospital of Capital Medical University.

**Table 1:** Clinical and demographic characteristics of the patient participants.

Patient No.	Gender	Age (years)	Analyzed electrodes	Hemisphere	Preoperative Medication (mg Q12h)
P01	F	19	40	R	Valproate 500; Lamotrigine 50; Levetiracetam 500
P02	M	25	39	R	Valproate 500; Oxcarbazepine 1200
P03	M	26	51	R	Valproate 400; Lamotrigine 100; Carbamazepine 400
P04	M	15	54	R	Valproate 500; Lamotrigine 100; Levetiracetam 500
P05	M	18	107	R	Levetiracetam 500; Oxcarbazepine 600
P06	M	25	104	L	Oxcarbazepine 450; Levetiracetam 500
P07	F	24	105	L	Oxcarbazepine 450; Levetiracetam 500
P08	M	24	68	L	Oxcarbazepine 600
P09	M	23	98	L	Oxcarbazepine 450; Valproate 500
P10	F	28	103	R	Oxcarbazepine 450

*M, male; F, female; L, left; R, right.*

**FIGURE 1:**
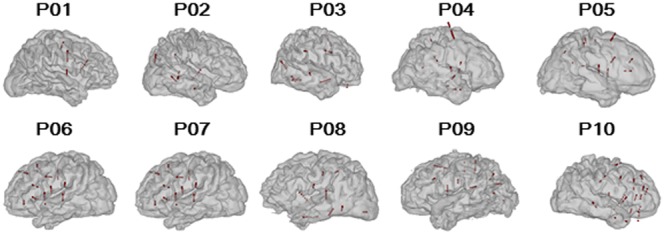
Reconstructed participant-specific brain models and locations of implanted electrodes. The black lines represent the paths of the depth electrodes and the red spheres represent the valid nodes (*n* = 769).

The participants were weaned from their antiepileptic medications during the monitoring period. The electrophysiological recordings for each participant were suspended for at least 4 h after a seizure to avoid the seizure-induced cortical suppression effect. The sEEG recordings were conducted only in a single hemisphere in each of the participants (six participants with right-hemisphere recordings, four participants with left-hemisphere recordings, **Figure 1**). To estimate the test-retest reliability, three participants (P04, P08, and P09) were tested twice on two different monitoring days.

### Apparatus and Stimuli

Gaussian wideband-noise stimuli were synthesized using the MATLAB version 2014b (MathWorks, Natick, MA, United States) at the sampling rate of 48 kHz with 16-bit amplitude quantization and low-pass filtered at 10 kHz. The duration of a noise-burst stimulus was 50 ms including the 5-ms linear ramp and damp. The acoustic stimulus was transferred using Creative Sound Blaster (Creative SB X-Fi Surround 5.1 Pro, Creative Technology Ltd., Singapore) and presented to participants with insert earphones (ER-3, Etymotic Research, Elk Grove Village, IL, United States) at the sound pressure level of 65 dB SPL. Calibration of the sound level was carried out with the Larson Davis Audiometer Calibration and Electroacoustic Testing System (AUDit and System 824, Larson Davis, Depew, NY, United States).

### Procedures and Electrophysiological Recordings

Each participant was reclining on a bed of a quiet room in the hospital during the experiment. To avoid unwanted effects of top-down modulation on neural responses, participants were instructed to watch a quiet TV show of their choice during the recording sessions. Thus, participants listened to the noise bursts passively without any task. For each of the two recording sessions, the noise burst was repeated 320 times. The inter-stimulus interval (ISI) between the two stimulus presentations was random between 900 to 1000 ms. The noise used in each trial was identical. The time required for each recording session was about 5–7 min. Three patients (P04, P08, and P09) were repeatedly recorded for the two recording sessions on different days.

Stereo-EEG responses were recorded using a Nicolet video-EEG monitoring system (Thermo Nicolet Corporation, United States), digitized at the rate of 1024 Hz and collected with a 0.05–200 Hz online bandpass filter. The sEEG electrodes were manufactured by Huake Hengsheng Medical Technology Co. Ltd., Beijing, China. The diameter of a depth electrode was 0.8 mm. The length of each node was 2 mm, which were spaced 1.5 mm apart from each other. The reference electrode was placed on the forehead. On a recording day, the impedance of all the recording electrode nodes was kept below 50 kΩ and the nodes whose impedances were higher than this value were excluded from analyses.

### Data Analyses

Three-dimensional brain images were reconstructed by pre-implantation MR images (T1 or contrast-enhanced) using BrainVoyager QX (Version 2.8, Brain Innovation B.V., Maastricht, Netherlands), and then were transferred into MATLAB data structures and further analyzed using NeuralAct toolbox (Kubanek and Schalk, 2015) in the MATLAB environment. Using BioImage software^1^, the original coordinates of electrode nodes were extracted from the images with the fusion between the pre-implantation MR and the post-implantation CT scans. The fused images were rotated to AC-PC plane, and were then registered to the standard brain ^2^ (Cox and Hyde, 1997; **RRID**: nif-0000-00259). The original coordinates were transferred to Talairach coordinates and used for identifying brain areas with Talairach Client^3^.

In each participant, epileptic foci had been identified before the recordings, and the electrode nodes which were located within the epileptic foci were excluded from data analyses. The pre-processing of electrophysiological data was conducted by the functions of the EEGLAB toolbox (Delorme and Makeig, 2004) in the MATLAB environment.

The long-term EEGs of each depth electrode were filtered by a band-pass filter (2–120 Hz) and segmented into epochs from -100 to 800 ms around the sound onset. The baseline correction was conducted by the time window from -100 to 0 ms before the sound onset. The epochs which contained more than ±1 mV potentials were rejected as artifacts. The remaining epochs were then averaged to obtain an event-related potential (ERP) for each electrode node. The evoked neural activities were calculated by the root mean square (RMS) of time windows of interest and then divided by the RMS of the pre-stimulus level (-50 to 0 ms). The time-frequency spectrum (Morlet wavelets approach, frequency step = 1 Hz) and GC analyses (time domain) were calculated using the Brainstorm toolbox^4^ (Tadel et al., 2011) in the MATLAB environment. Mother wavelet parameters were set to full width half maximum value of 3 s for the Gaussian kernel at a center frequency of 1 Hz.

The GC is considered from X to Y (i.e., X→Y) if including past values of X and Y (i.e., full model) provides more information about future values of Y compared to when only the past values of Y (i.e., restricted model) are considered (Seth, 2010). Here, X or Y are time series representing sound-evoked (broadband) potentials for a particular electrode location and participant. Note that electrode nodes only from the same person are paired and used for GC analyses. The higher GC value represents a stronger interaction from X to Y. To assess the statistical significance (*p*-value) of the GC value between two electrodes X→Y, we tested the null hypothesis (i.e., the full model did not fit the data better than the restricted model) using the F-statistic. Only the significant GC values were entered into further analyses.

Statistical analyses were performed with IBM SPSS Statistics 20 (SPSS Inc., Chicago, Illinois 60606). To analyze dynamic changes in either amplitudes or GC values of sound-evoked responses, (within-subjects) repeated-measures analyses of variance (ANOVAs), *t*-tests, Pearson correlation, and Bonferroni *post hoc* tests were conducted. The *vegan* package of R (version 2.15.0) was used to test correlations among GC matrixes (Mantel *r* tests). The null-hypothesis rejection level was set at 0.05.

## Results

The electrode nodes with over threshold impedances (>50 kΩ), artifacts, and/or within epilepsy foci were excluded from data analyses. Data obtained from 769 electrode nodes in 10 participants (**Figure 1**) were used for further analyses (the number of electrodes for each patient: P01, *n* = 40; P02, *n* = 39; P03, *n* = 51; P04, *n* = 54; P05, *n* = 107; P06, *n* = 104; P07, *n* = 105; P08, *n* = 68; P09, *n* = 98; and P10, *n* = 103).

### Noise-Burst-Evoked Potentials in the Auditory Cortex and Non-auditory Cortices

Using the Talairach coordinates, all the 769 depth electrodes were located in the following brain areas: the AC (*n* = 61), middle frontal gyrus (MFG, *n* = 48), inferior frontal gyrus (IFG, *n* = 23), precentral gyrus (*n* = 64), postcentral gyrus (PoG, *n* = 27), inferior parietal lobule (IPL, *n* = 35), superior temporal gyrus (*n* = 26), middle temporal gyrus (MTG, *n* = 37), inferior temporal gyrus (ITG, *n* = 11), parahippocampal gyrus (ParaHipp, *n* = 35), insula (*n* = 33), cingulate gyrus (*n* = 72), fusiform gyrus (*n* = 31), and precuneus (*n* = 43). Examples of original ERPs and time-frequency analyses see in Supplementary Figures S1, S2. The electrode nodes that were located in subcortical structures were not analyzed. The electrode distribution in individual participants is presented in **Table 2**.

**Table 2:** Electrodes distribution of individual participants.

	Brain areas											
Patient No.	IFG	MFG	PreG	PoG	IPL	ITG	MTG	STG	AC	Insula	ParaHipp	Other electrodes
P01	0	0	11	3	0	0	0	2	0	2	0	22
P02	0	0	0	0	8	2	9	0	6	0	4	10
P03	2	1	1	0	7	2	9	3	0	0	4	22
P04	0	0	12	1	0	0	1	6	9	3	10	12
P05	9	14	9	10	8	0	0	2	6	7	0	42
P06	0	3	8	1	10	4	7	4	11	2	9	45
P07	7	12	11	3	2	0	0	1	17	7	0	45
P08	0	0	0	3	0	3	4	4	5	0	5	44
P09	0	1	7	0	0	0	2	1	0	9	0	78
P10	5	17	5	6	0	0	5	3	7	3	3	49
*Total*	*23*	*48*	*64*	*27*	*35*	*11*	*37*	*26*	*61*	*33*	*35*	*369*

*This table shows the electrodes distribution over the 11 significantly activated brain areas. Other electrodes includes the ones located in subcortical areas and electrodes located in cingulate gyrus, fusiform gyrus, and precuneus, which were not significantly activated by the noise burst.*

The dynamic neural activities of recorded cortical regions were calculated as the relative amplitude indices (using RMS) in the five time windows after the sound onset (i.e., 0–50, 50–100, 100–150, 150–200, and 200–250 ms) with the baseline level within the time window from -50 to 0 ms. Repeat-measured *t*-tests between the RMS in each time windows of interest and the RMS from the baseline time window were conducted (with Bonferroni correction).

**FIGURE 2:**
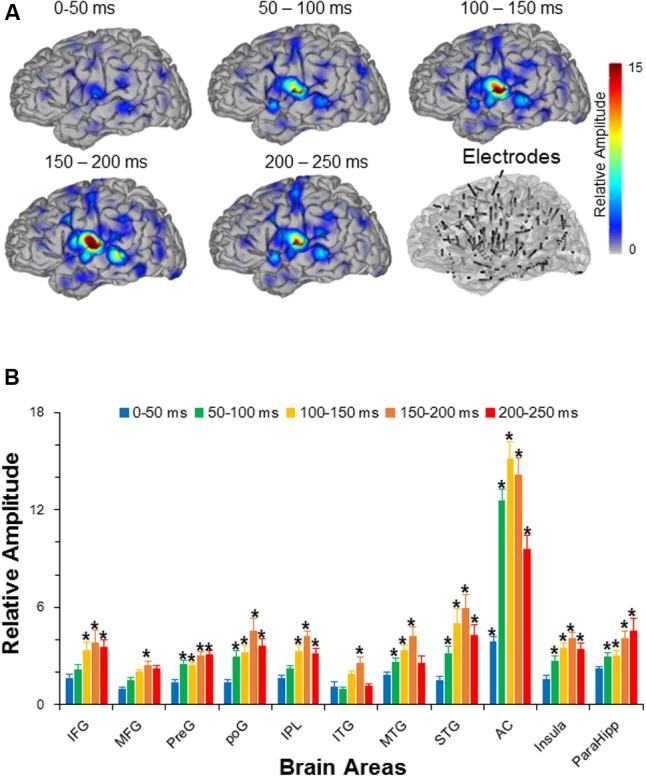
**(A)** The grand averaged activation maps calculated by relative amplitudes within the five time windows after the sound onset. Locations of all the depth electrodes are transferred into the left hemisphere for exhibition. The bottom right panel shows the electrode node distribution on a standard brain. **(B)** Comparisons in relative amplitudes across the 11 significantly activated brain areas within the five time windows after the sound onset. The root mean squares in the five time windows (0–50, 50–100, 100–150, 150–200, and 200-250 ms after the sound onset) were divided by the root mean square in the time window from –50 to 0 ms to obtain relative amplitudes, respectively. *AC*, auditory cortex; *PreG*, precentral gyrus; *STG*, superior temporal gyrus; *poG*, postcentral gyrus; *IPL*, inferior parietal lobule; *IFG*, inferior frontal gyrus; *MFG*, middle frontal gyrus; *MTG*, middle temporal gyrus; *ParaHipo*, para-hippocampus; *ITG*, inferior temporal gyrus. *^∗^ p <*0.05.

The results showed that there were in total 11 out of 14 recorded brain areas (MFG, IFG, preG, poG, IPL, ITG, MTG, STG, AC, ParaHipp, and Insula) that were significantly activated by the noise burst within at least one of five time windows. The noise-burst-evoked potentials in the AC were markedly different from those in the non-AC areas: Potentials in the AC were larger, occurred earlier, and lasted longer than those in the non-AC areas. As shown in **Figure 2A**, the activation cores distributed around the Sylvian fissure, where the AC was located.

Independent *t*-tests confirmed that the relative amplitudes of AC were significantly larger than those of the non-AC areas in each of the five time windows (all Bonferroni corrected *p* < 0.05). In addition, only the evoked amplitudes of the AC, but not those of the non-AC areas, were significant in the first time window (0–50 ms) after the sound onset (Bonferroni corrected *p* < 0.05, repeat-measured *t*-tests).

**FIGURE 3:**
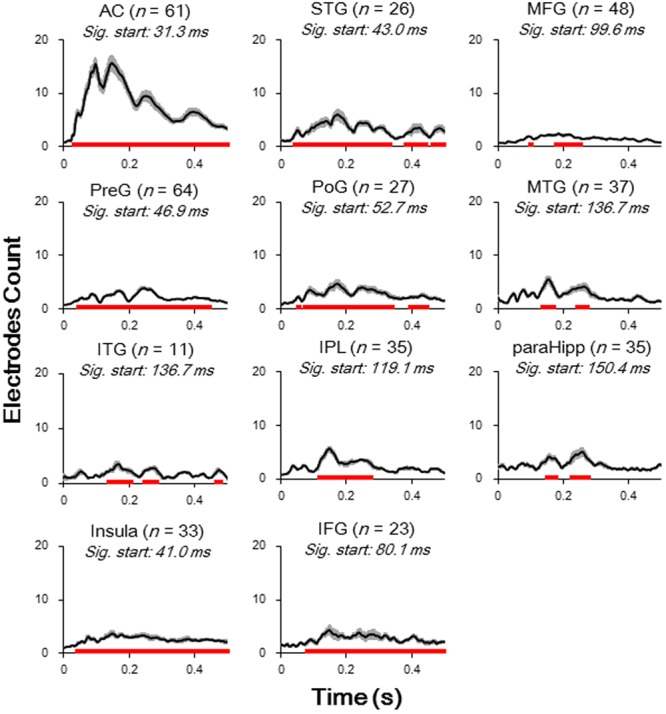
Averaged waveform (absolute value) for each of significantly activated brain areas. Student *t*-tests against baseline (–50 to 0 ms before sound onset) with Bonferroni correction were conducted to decide the significance. *Black lines*, mean value; *Gray areas*, standard error; *Red bars*, significant temporal area; *Sig. start*, the time point when the significance started to occur; *AC*, auditory cortex; *PreG*, precentral gyrus; *STG*, superior temporal gyrus; *poG*, postcentral gyrus; *IPL*, inferior parietal lobule; *IFG*, inferior frontal gyrus; *MFG*, middle frontal gyrus; *MTG*, middle temporal gyrus; *ParaHipp*, para-hippocampus; *ITG*, inferior temporal gyrus.

The relative amplitudes for the following brain areas, including the preG, poG, STG, ParaHipp, and insula, started to be significant in the second (50–100 ms) time window (all Bonferroni corrected *p* < 0.05, repeat-measured *t*-tests). The relative amplitudes for the IFG and IPL started to be significant in the third time window (100–150 ms), and the relative amplitudes for the MFG and ITG were significant only in fourth time window (150–200 ms) (all Bonferroni corrected *p* < 0.05, repeat-measured *t*-tests) (**Figures 2B, 3**). For longer temporal window analyses see in Supplementary Figure S3.

**FIGURE 4:**
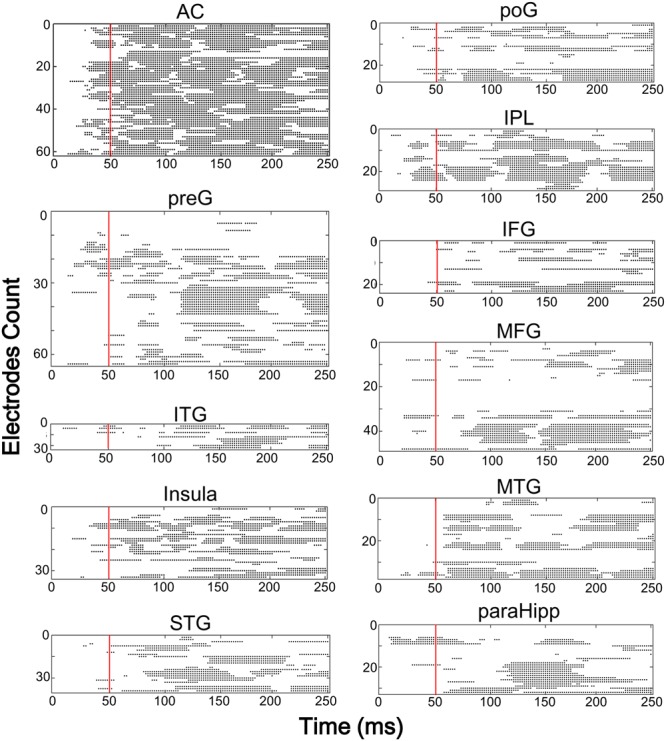
The results of single trial statistical analysis within each electrode, respectively. Each line represents the significance across time and each black dot represented this time point was significantly different with the baseline. *Red lines*, time points at 50 ms. *AC*, auditory cortex; *PreG*, precentral gyrus; *STG*, superior temporal gyrus; *poG*, postcentral gyrus; *IPL*, inferior parietal lobule; *IFG*, inferior frontal gyrus; *MFG*, middle frontal gyrus; *MTG*, middle temporal gyrus; *ParaHipp*, para-hippocampus; *ITG*, inferior temporal gyrus.

Furthermore, latencies of these 11 (out of 14) brain areas to the noise-burst stimulus were extracted from the first peak response (with either positive or negative polarity). Single-trial statistical analyses for each electrode were conducted. As shown in **Figure 4**, the first peak was defined as the first significant data point while the latency was defined as the time from sound onset to the first peak. The latencies of the AC (*M* = 52.3, *SD* = 25.3, here and below in ms) were significantly shorter than all those of the non-AC areas, including the preG (*M* = 84.4, *SD* = 42.9), ITG (*M* = 88.6, *SD* = 27.3), insula (*M* = 91.2, *SD* = 43.1), STG (*M* = 92.7, *SD* = 38.6), poG (*M* = 97.4, *SD* = 45.2), IPL (*M* = 104.1, *SD* = 47.6), IFG (*M* = 104.6, *SD* = 43.8), MFG (*M* = 107.6, *SD* = 32.0), MTG (*M* = 118.7, *SD* = 49.6), and ParaHipp (*M* = 127.6, *SD* = 44.8) (all Bonferroni corrected *p* < 0.05, independent *t*-tests).

### Intrinsic and Extrinsic Networks of the AC

**FIGURE 5:**
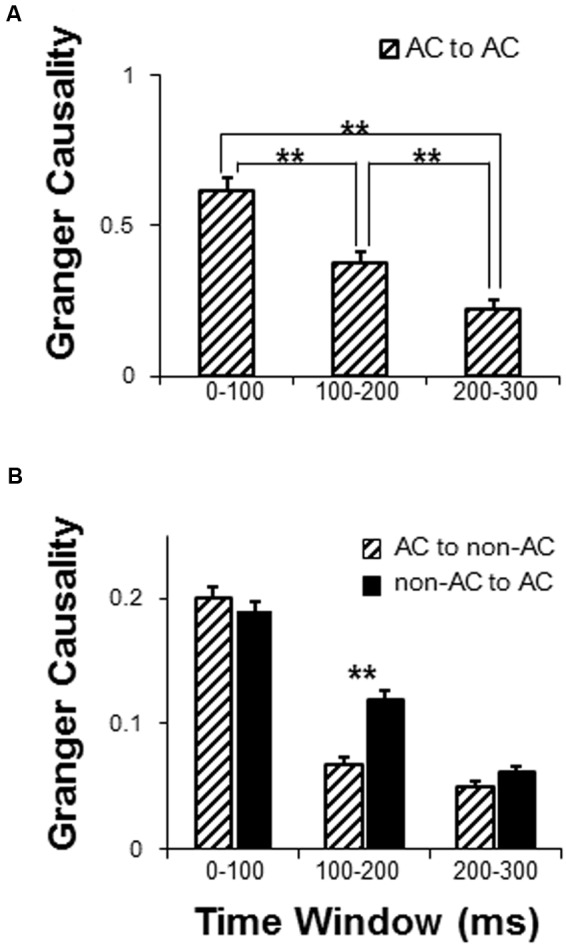
Comparison of dynamic networks with in the following three time windows: 0–100, 100–200, and 200–300 ms. **(A)** Granger causality (GC) analyses of the auditory cortex local network. **(B)** GC analyses of functionally connected networks of the AC. *AC*, auditory cortex. *^∗∗^ p <* 0.01.

Granger causality analyses were used to estimate the sound-evoked functional networks including the local network within the AC (electrode-node pairs were located within the AC area) and external networks interacting with the AC (electrode-node pairs between the AC and non-AC areas) for the following three time windows after the sound onset (0–100, 100–200, and 200–300 ms). There were in total 601 AC-AC electrode pairs that were entered into analyses. Generally, the results of one-way repeated-measure ANOVA showed that the GC value within the AC (iFC) gradually attenuated with time (*F*_2,1200_ = 56.14, *p* < 0.001; *post hoc*: all *p* < 0.01, with *Bonferroni* adjustment) (**Figure 5A**).

There were in total 4108 AC to non-AC electrode pairs that were entered into analyses. For the external network of the AC, a 2 (connectivity direction: AC to non-AC, non-AC to AC) by 3 (time window: 0–100, 100–200, and 200–300 ms) two-way repeated-measures ANOVA showed that the main effect of connectivity direction was significant (*F*_1,4108_ = 9.58, *p* = 0.002), the main effect of time window was significant (*F*_2,8216_ = 228.05, *p* < 0.001), and the interaction was significant (*F*_2,8216_ = 10.83, *p* < 0.001) (**Figure 5B**). *Post hoc* results showed that the GC values significantly attenuated across time windows for both the AC to non-AC direction and the non-AC to AC direction (all *p* < 0.05, with *Bonferroni* adjustment). In addition, the connectivity-direction effect was significant for the time window of 100–200 ms (*p* < 0.001, with *Bonferroni* adjustment) but not for the time windows of 0–100 ms and 200–300 ms (*p* = 0.420 and *p* = 0.091, respectively, with *Bonferroni* adjustment) (**Figure 5B**).

A 2 (connectivity direction: from AC to non-AC, from non-AC to AC) by 3 (time window: 0–100, 100–200, and 200–300 ms) two-way repeated-measures ANOVA was conducted for each of the 10 non-AC areas. As shown in **Table 3**, the main effect of time window was significant for each of the non-AC areas (all *p* < 0.05), indicating that the strength of connectivity with the AC generally decayed with time. The main effect of connectivity direction was significant for five non-AC areas, including the MTG, ITG, ParaHipp, IPL, and preG (all *p* < 0.05). The interaction effect was significant for five non-AC areas, including the STG, ITG, IPL, preG, and MFG.

**Table 3:** Statistical results of granger causalities between the auditory cortex (AC) and non-auditory cortices.

Modulation (to/from AC)	Main effects of ANOVAs						Modulated direction *post hoc* tests					
	Time window		Modulation direction		Interaction effect		0–100 ms		100–200 ms		200–300 ms	
STG	***F*_2,232_ = 17.893**	***p* < 0.001**	*F*_1,116_ = 3.710	*p* = 0.057	***F*_2,232_ = 3.297**	***p* = 0.039**	**Top-down**	***p* = 0.016**	N.S.	*p* = 0.863	N.S.	*p*= 0.371
MTG	***F*_2,388_ = 7.705**	***p* = 0.001**	***F*_1,194_ = 14.274**	***p* < 0.001**	*F*_2,388_ = 0.608	*p* = 0.545	**Top-down**	***p* = 0.028**	N.S.	*p* = 0.069	**Top-down**	***p*= 0.012**
ITG	***F*_2,140_ = 13.458**	***p* < 0.001**	***F*_1,70_ = 4.986**	***p* = 0.029**	***F*_2,140_ = 3.916**	***p* = 0.022**	**Bottom-up**	***p* = 0.017**	N.S.	*p* = 0.655	N.S.	*p*= 0.961
Insula	***F*_2,376_ = 14.165**	***p* < 0.001**	*F*_1,188_ = 2.451	*p* = 0.119	*F*_2,376_ = 0.269	*p* = 0.764	N.S.	*p* = 0.811	N.S.	*p* = 0.256	**Top-down**	***p*= 0.037**
ParaHipo	***F*_2,516_ = 20.994**	***p* < 0.001**	***F*_1,258_ = 3.968**	***p* = 0.047**	*F*_2,516_ = 1.199	*p* = 0.302	N.S.	*p* = 0.930	**Top-down**	***p* = 0.001**	N.S.	*p*= 0.062
IPL	***F*_2,382_ = 20.282**	***p* < 0.001**	***F*_1,191_ = 16.915**	***p* < 0.001**	***F*_2,382_ = 12.854**	***p* < 0.001**	**Bottom-up**	***p* < 0.001**	N.S.	*p* = 0.824	N.S.	*p*= 0.279
preG	***F*_2,834_ = 9.006**	***p* < 0.001**	***F*_1,417_ = 5.398**	***p* = 0.021**	***F*_2,834_ = 3.718**	***p* = 0.025**	N.S.	*p* = 0.455	**Top-down**	***p* < 0.001**	N.S.	*p*= 0.678
poG	***F*_2,254_ = 15.433**	***p* < 0.001**	*F*_1,127_ = 0.488	*p* = 0.486	*F*_2,254_ = 1.936	*p* = 0.146	N.S.	*p* = 0.305	N.S.	*p* = 0.210	N.S.	*p*= 0.097
MFG	***F*_2,710_ = 17.251**	***p* < 0.001**	*F*_1,355_ = 2.375	*p* = 0.124	***F*_2,710_ = 3.508**	***p* = 0.030**	N.S.	*p* = 0.386	**Top-down**	***p* = 0.006**	N.S.	*p*= 0.071
IFG	***F*_2,306_ = 9.790**	***p* < 0.001**	*F*_1,153_ = 0.032	*p* = 0.857	*F*_2,306_ = 0.040	*p* = 0.960	N.S.	*p* = 0.917	N.S.	*p* = 0.734	N.S.	*p*= 0.882

*Boldface characters represent the significant effects (*p* < 0.05).*

**FIGURE 6:**
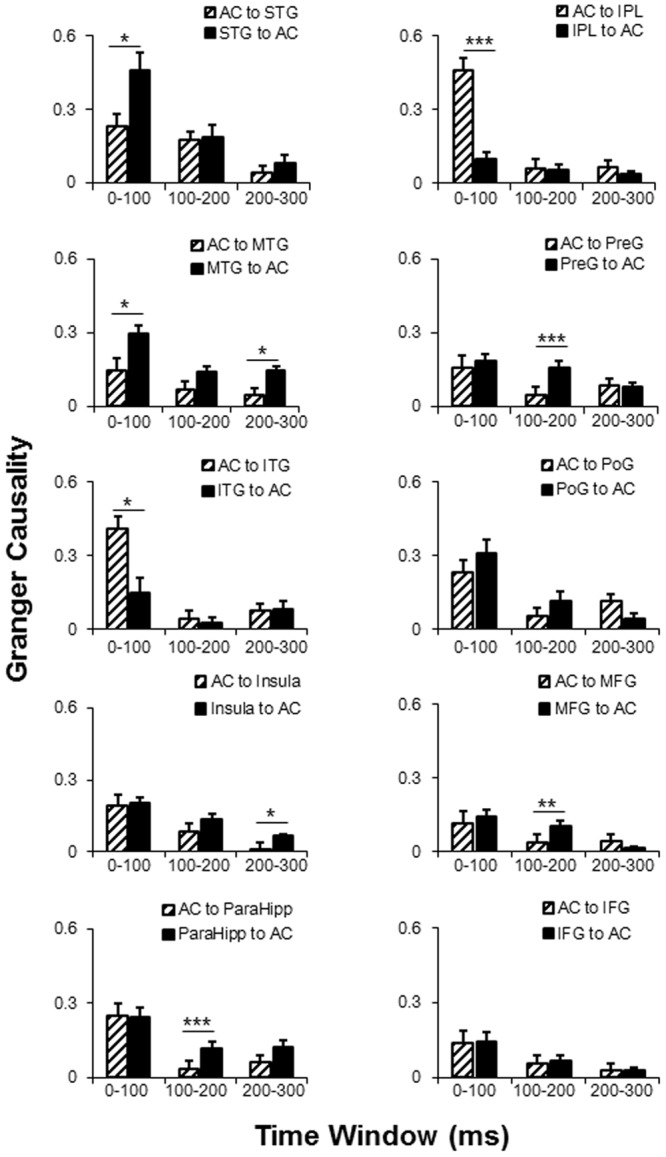
Comparisons of the dynamic GC analyses between AC and the 10 non-auditory cortical areas. *AC*, auditory cortex; *PreG*, precentral gyrus; *STG*, superior temporal gyrus; *poG*, postcentral gyrus; *IPL*, inferior parietal lobule; *IFG*, inferior frontal gyrus; *MFG*, middle frontal gyrus; *MTG*, middle temporal gyrus; *ParaHipo*, para-hippocampus; *ITG*, inferior temporal gyrus. *^∗^ p <* 0.05; *^∗∗^ p* < 0.01; *^∗∗∗^ p* < 0.001.

*Post hoc* tests of the connectivity-direction effect showed that (1) in the 0–100 ms time window, the AC mainly sent bottom-up outputs to the ITG and IPL, and received top-down inputs from the STG and MTG (all *p* < 0.05, with *Bonferroni* adjustment); (2) in the 100–200 ms time window, the AC was mainly top-down modulated by the ParaHipp, preG, and MFG (all *p* < 0.05, with *Bonferroni* adjustment); (3) in the 200–300 ms time window, the AC was mainly top-down modulated by the MTG and insula (all *p* < 0.05, with *Bonferroni* adjustment); (4) within any time windows, no significant top-down or bottom-up connectivity effect was found between the AC with either the poG or the IFG (all *p* > 0.05, with *Bonferroni* adjustment) (**Figure 6** and **Table 3**).

### Interactions between Intrinsic Connectivity and Extrinsic Connectivity of the AC

**FIGURE 7:**
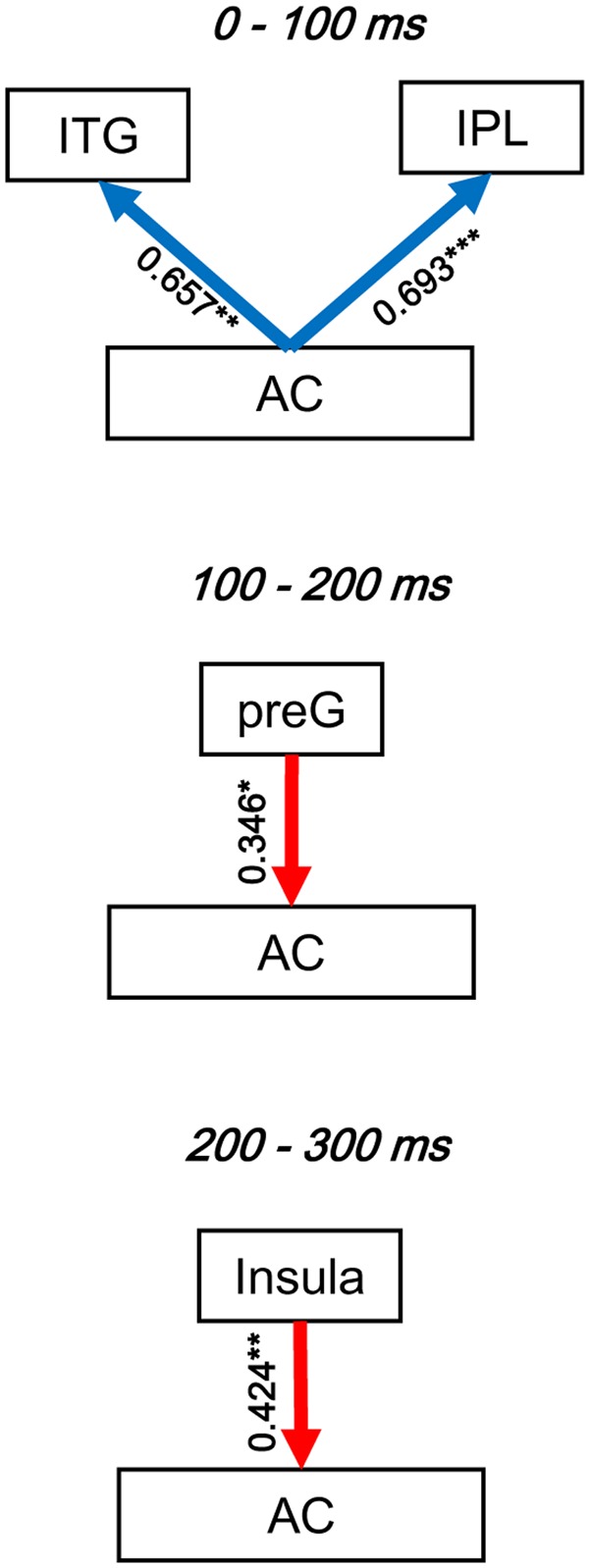
Illustration of the dynamic interactions between the AC local network and functionally connected networks of the AC. The correlation coefficients were obtained by Pearson correlation tests. *Blue arrows*, bottom-up modulation from the AC to non-auditory areas. *Red arrows*, top-down modulation from non-auditory areas to the AC. *Bold arrows*, significant correlation. *^∗^ p* < 0.05; *^∗∗^ p*< 0.01; *^∗∗∗^ p* < 0.001.

To evaluate the dynamic interactions between iFC and eFC of the AC, Pearson correlation tests were conducted between GCs of iFC of the AC and GCs of the significantly modulated non-AC areas within each of the three time windows (0–100, 100–200, and 200-300 ms). In each of the pairwise correlation tests, the GC from AC-Node1 to AC-Node 2 and the GC from AC-Node1 to non-AC-Node1 (for example, ITG) were one-to-one matched. Note that the iFC pair that could not match any eFC was excluded from the correlation test. The results showed that in the 0–100 ms time window, iFC of the AC was significantly correlated with bottom-up eFC from the AC to both the ITG (*r*_16_ = 0.657, *p*= 0.004) and the IPL (*r*_33_ = 0.693, *p*< 0.001). In the 100–200 ms time window, iFC of the AC was significantly correlated with eFC from the preG to the AC (*r*_43_ = 0.346, *p*= 0.021; adjusted by *Bonferroni* approach). In the 200–300 ms time window, iFC of the AC was significantly correlated with eFC from the insula to AC (*r*_43_ = 0.424, *p*= 0.004; adjusted by *Bonferroni* approach, **Figure 7** and **Table 4**).

**Table 4:** Pearson correlation coefficients between extrinsic networks and the local AC network.

	Correlation coefficients				
	local AC network		AC activity	
***0–100 ms***				
AC to ITG	***r_16_* = 0.657**	***p* = 0.004**	*r_16_*= 0.314	*p* = 0.220
AC to IPL	***r_33_* = 0.693**	***p* < 0.001**	*r_33_* = 0.280	*p* = 0.109
MTG to AC	*r_32_* = -0.062	*p* = 0.731	*r_32_* = -0.151	*p* = 0.400
STG to AC	*r_43_* = 0.026	*p* = 0.866	*r_43_* = -0.134	*p* = 0.384
***100–200 ms***				
preG to AC	***r_43_* = 0.346**	***p* = 0.021**	*r_43_* = -0.031	*p* = 0.845
MFG to AC	*r_34_* = 0.101	*p* = 0.564	*r_34_* = -0.157	*p* = 0.137
ParaHipo to AC	*r_32_* = 0.229	*p* = 0.199	*r_32_* = -0.080	*p* = 0.657
***200–300 ms***				
MTG to AC	*r_32_* = 0.259	*p* = 0.145	*r_32_* = 0.060	*p* = 0.741
Insula to AC	***r_43_* = 0.424**	***p* = 0.004**	*r_43_* = -0.174	*p* = 0.257

*Boldface characters represent the significant effects (*p* < 0.05).*

### Test-Retest Reliability

**FIGURE 8:**
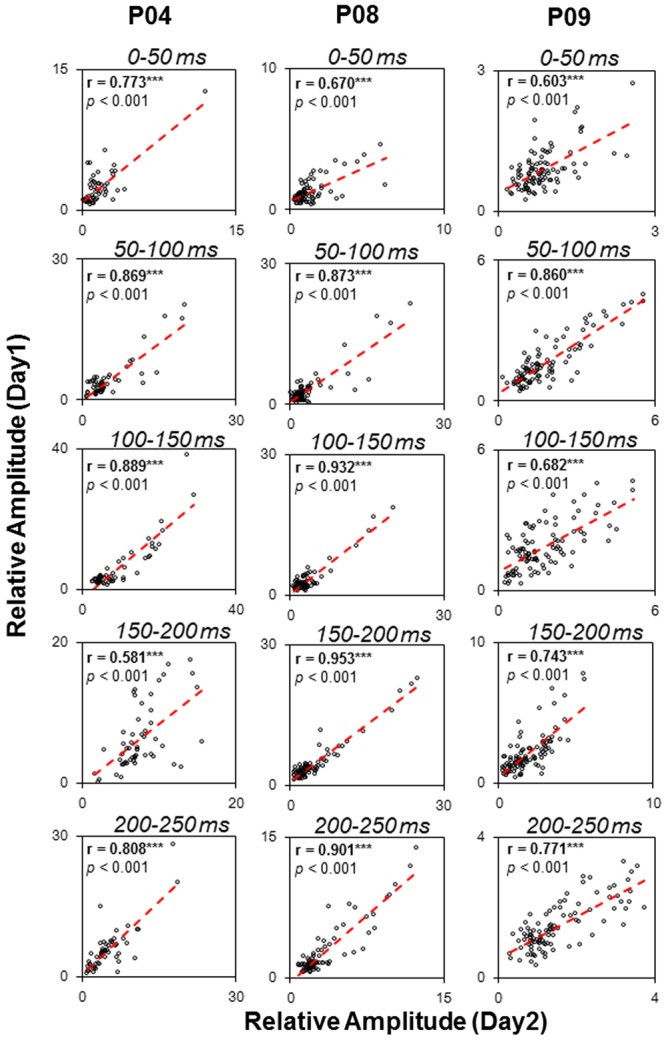
Test-retest reliability (Pearson correlation tests) of noise-burst-evoked activities in three participants (P04, P08, and P09). *^∗∗∗^ p* < 0.001.

To examine the test-retest reliability of the auditory functional networks obtained by the sEEG procedure used in this study, three participants (P04, P08, and P09) were recorded on two different days. The consistency of the two recordings were examined by correlation analyses. Pearson correlations between the relative amplitudes in the five temporal windows (0–50, 50–100, 100–150, 150–200, and 200–250 ms) on the first recording day and those on the second recording day were remarkably significant (all *p* < 0.05, with *Bonferroni* adjustment, **Figure 8**), indicating that the noise burst-evoked potentials in these brain areas exhibited a high consistence.

**FIGURE 9:**
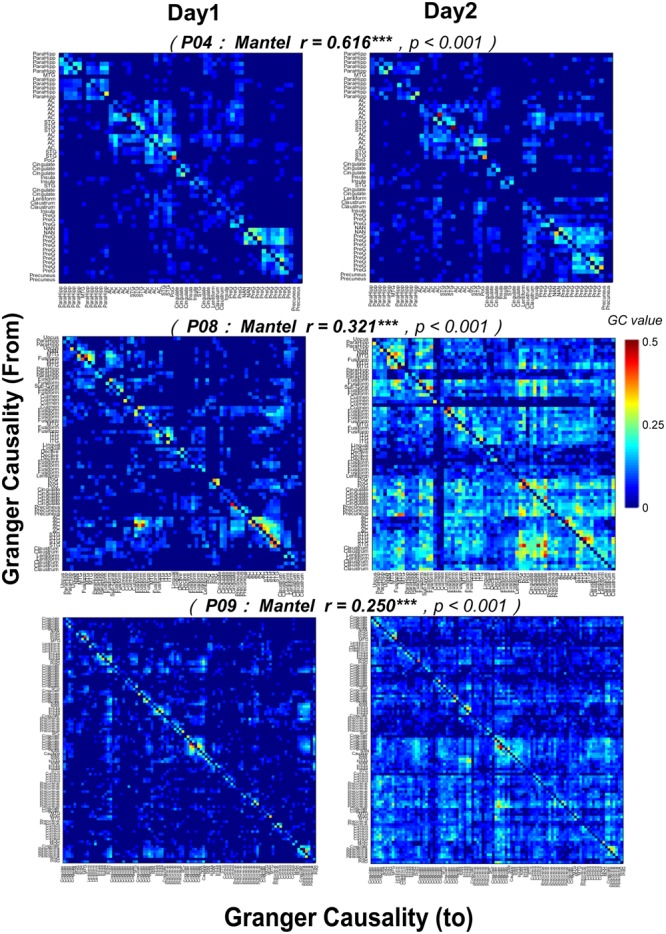
Test-retest reliability (Mantel *r* tests) of noise-burst-evoked GC networks in three participants (P04, P08, and P09). Each of the colorful pixel represents a certain GC value from one electrode to another. The colorful pixel on the diagonal, which represents the GC from a certain electrode to itself and is irrelevant to cross-electrodes analyses, is kept to be zero. The total number of electrodes in each of three participants was 54 (P04), 68 (P08), and 98 (P09), respectively. *^∗∗∗^ p*< 0.001.

Furthermore, the correlations between the noise-burst-evoked GC network matrixes (from or to the AC, 0–300 ms) of one participant on the first recording day and those of the same participant on the second recording day were significant (Mantel *r* tests, all *p* < 0.05, with *Bonferroni* adjustment, **Figure 9**), indicating that the procedure for estimating auditory function networks had a high test-retest reliability.

## Discussion

This study confirms previous reports that in humans both the AC and higher-order cortices can be activated by a sound burst under passive listening conditions (Engelien et al., 1995; Cheung et al., 2016). Utilizing the intracranial sEEG recording method with both high spatial and high temporal resolutions, this study reveals not only dynamic patterns of sound-evoked activation in the AC and some non-auditory cortices, but also interactions between the AC and the higher-order non-auditory cortices.

### Earlier and Later Cortical Processing of Auditory Signals

According to the prevalent view, ascending sensory signals reach modality-specific sensory cortices first and then propagate to other higher-order cortices (Pickles, 1982). For example, some scalp-ERP studies have shown that the lower-order AC contributes to the earlier potentials (e.g., P50 and N100) to sound stimulation and the higher-order non-auditory cortices contribute to the later potentials (e.g., P200) (Picton et al., 1974; Scherg et al., 1989). The results of this study indicate that the sound-evoked first-peak potentials with latencies shorter than 50 ms occur only in the AC. Also, the relative amplitudes of ERPs in the AC are consistently larger than those in the non-auditory cortices. Some non-auditory cortices, including the PreG, ITG, insula, STG, and PoG exhibit the first-peak potentials with latencies between 50 and 100 ms. The non-auditory cortices that exhibit the first-peak potentials with latencies longer than 100 ms include the IPL, IFG, MFG, MTG, and ParaHipp. Since these higher-order non-auditory cortices respond to sound-stimulus signals at different time windows, it is of interest to investigate how these non-auditory cortices contribute differentially to the processing of sound signals.

Note that since both the PreG and insula also receive direct axonal projections from the auditory thalamus (Burton and Jones, 1976; Mesulam and Mufson, 1985; Henschke et al., 2015), the first-peak potentials of the PreG and insula may also be driven by the auditory thalamus.

The results of GC analyses of this study suggest that the neural networks with the time window within the first 100 ms after the sound onset show a directional complexity of eFC of the AC: Both bottom-up and top-down signal flow occur between the AC and some non-auditory cortices in this early time window (bottom-up: from the AC to the ITG and IPL; top-down: from the MTG and STG to the AC). Previous neuroimaging studies have suggested that the STG, MTG, and ITG belong to the ventrolateral cortical pathway for sound discrimination (Waberski et al., 2001) and speech perception (Binder et al., 1996, 2000). On the other hand, as a key structure in the dorsal cortical pathway for spatial processing, the IPL is essential for auditory spatial perception (Heilman and Valenstein, 1972; Weeks et al., 1999). The bottom-up eFC from the AC to the ITG and that to the IPL indicate that the sound representation in the AC initializes the sound representation in both the ventrolateral cortical pathway for sound discrimination and the sound representation in the dorsal cortical pathway for spatial processing. The top-down eFC from the MTG/STG to the AC may enhance the sound discrimination process. Thus, further studies in the future should be carried out to examine how the bi-directional eFC between the AC and the higher-order non-auditory cortices plays a role in establishing auditory detection, identification, localization, and/or speech analyses.

### Functional Interaction between Intrinsic Connectivity and Extrinsic Connectivity of the Auditory Cortex

The AC receives auditory inputs from both cortical and subcortical structures that are implicated in auditory processing, attention, and learning (Kilgard and Merzenich, 1998; Eliades and Wang, 2003; Polley et al., 2006; Froemke et al., 2007; Budinger et al., 2008; Letzkus et al., 2011). It is of interest to know whether fluctuations of the sound-evoked local activities over time in the AC is associated with these auditory functions.

The AC contains neuronal circuits at multiple scales (Mitani et al., 1985; Barbour and Callaway, 2008; Lee and Winer, 2011; Winer, 2011), forming a hub that both extracts and integrates ascending auditory spectro-temporal features (Griffiths and Warren, 2002; Eggermont, 2007; Gourévitch and Eggermont, 2010; Atencio and Schreiner, 2016). Although previous studies have suggested that iFC within the AC is functionally related with the pitch perception (Albouy et al., 2013), auditory stream segregation (Giani et al., 2015), and integration of different auditory features (Tardif and Clarke, 2001), it is still not clear how iFC of the AC interacts with eFC of the AC for achieving this functions.

This study reveals that although the GC strength of iFC of the AC gradually decays, it does not reach the baseline level within the period of 100–200 ms following the sound onset (up to 150 ms after the sound offset). In the same time period, the information flow from the non-auditory cortices to the AC are significantly stronger than those from the AC to the non-auditory cortices. Thus, the non-auditory cortices may have a strong top-down modulation on the local AC network.

Cognitive functions are based on interactions of various networks in the brain (Bressler and Menon, 2010). The temporal dynamic of the interactions between iFC and eFC of the AC with non-auditory cortices has not been addressed in the literature. The current study for the first time reveals that iFC of the AC is positively correlated with not only the earlier bottom-up flow from the AC to the ITG and IPL, but also the later top-down flow from the PreG and insula to the AC.

It has been known that motor cortices and sensory cortices are not functionally separated. In fact, both sensory and motor cortices are engaged at the same time during perception (Gallese et al., 1996; Wilson and Moss, 2004; Tkach et al., 2007; Cogan et al., 2014). Particularly, listening to speech sounds also evokes robust neural activities in motor cortices (Wilson et al., 2004; Pulvermüller et al., 2006; Edwards et al., 2010; Cogan et al., 2014). Also, motor cortical areas have bidirectional connections with the STG (Zatorre et al., 2007; Rauschecker and Scott, 2009; Cheung et al., 2016), AC (Nelson et al., 2013; Schneider et al., 2014), and the auditory thalamus (Henschke et al., 2015). Thus, the AC integrates both bottom-up signals concerning sound features and top-down signals concerning impending movements and motor planning (Nelson and Mooney, 2016). The modulation from the motor area to the local auditory network revealed by this study reflects a feedback in sensory-motor integration (Hickok et al., 2011).

Moreover, anatomically, the insula receives afferents from the auditory thalamus (Burton and Jones, 1976; Mesulam and Mufson, 1985) and also has connections with the AC (Mesulam and Mufson, 1985) and other association cortices (Augustine, 1985). The insula participates in several auditory processes (Bamiou et al., 2003). Lesions of the bilateral insular lead to auditory agnosia (Fifer, 1993; Habib et al., 1995). Previous studies have also shown that the insula is essential to auditory decision processing (Binder et al., 2004), auditory temporal processing (Ackermann et al., 2001), and sound movement detection (Lewis et al., 2000). The modulation of the AC by the insula will be an interesting issue in future studies.

It should be noted that although the intracranial EEG recording method provides an opportunity to obtaining electrophysiological responses of neuron populations, it bears some shortcomings such as that the electrode placements are sparse compared to the total brain size and each patient has a different coverage of different brain areas. Particularly note that GC analysis may only reveal linear relationships between brain areas, and some non-linear relationships may be either approximated or missed. One important issue that should be addressed in the future is whether interactions between iFC and eFC of the AC are critical for target-sound (such as target-speech) listening under “cocktail-party” conditions.

## Conclusion

This study reveals three types of functional connectivity that declines over time after the sound onset: (1) iFC within the AC, (2) bottom-up eFC from the AC to non-auditory cortices, and (3) top-down eFC from non-auditory cortices to the AC. Shortly after the sound onset, iFC of the AC drives out-going eFC to the ITG in the ventral cortical pathway and out-going eFC to the IPL in the dorsal pathway. And then, iFC of the AC reflects top-down eFC from the motor region (PreG) and that from the insula. Interactions between iFC and eFC of the AC following the sound stimulation may be fundamental not only to auditory sensory memory and object formation, but also to integration across sensory, perception, attention, motor, emotion, and executive processes.

## Author Contributions

ZC, QW, YaG, and JW: Experimental design, experiment set up, experiment conduction, data analyses, figure/table construction, and paper writing. MW and PT: Experimental design, data analyses, and paper writing. YuG: Experimental design and paper writing. JZ: Experimental design, experiment set up, and paper writing. TL, GL, and LL: Experimental design, figure/table construction, and paper writing.

## Conflict of Interest Statement

The authors declare that the research was conducted in the absence of any commercial or financial relationships that could be construed as a potential conflict of interest.

## Footnotes

1 http://bioimagesuite.yale.edu

2 http://afni.nimh.nih.gov/

3 http://www.talairach.org/client.html

4 http://neuroimage.usc.edu/brainstorm/
