# Supplementary material for: Dynamic Correlations between Intrinsic Connectivity and Extrinsic Connectivity of the Auditory Cortex in Humans
Source: Front Hum Neurosci. 2017 Aug 11;11:407. doi: 10.3389/fnhum.2017.00407 (PMC5554526; doi:10.3389/fnhum.2017.00407)
Supplement: Supplementary file 1 [file Presentation_1.PDF]

Supplementary Information

Dynamic Correlations between Intrinsic Connectivity and Extrinsic Connectivity of the Auditory Cortex in Humans

Zhuang Cui[a,b,#], Qian Wang[a,c,#], Yayue Gao[c], Jing Wang[a], Mengyang Wang[a], Pengfei Teng[a], Yuguang Guan[a], Jian Zhou[a], Tianfu Li[a,d], Guoming Luan[a,d,*], Liang Li[c,d,*]

[a]Beijing Key Laboratory of Epilepsy, Epilepsy Center, Department of Functional Neurosurgery, Sanbo Brain Hospital, Capital Medical University, Beijing, China 100093

[b]Beijing Hospital, Beijing, China 100730

[c]School of Psychological and Cognitive Sciences and Beijing Key Laboratory of Behavior and Mental Health, Key Laboratory on Machine Perception (Ministry of Education), Peking University, Beijing, China 100080

[d]Beijing Institute for Brain Disorders, Beijing, China 100069

[#]These two authors contributed equally to this work

***Correspondence:**

Guoming Luan, M.D., Ph.D.

Beijing Key Laboratory of Epilepsy, Epilepsy Center, Department of Functional Neurosurgery, Sanbo Brain Hospital, Capital Medical University, Beijing, China 100093

E-mail: luangm3@163.com

Liang Li, Ph.D.

School of Psychological and Cognitive Sciences, Peking University, Beijing, China 100080

E-mail: liangli@pku.edu.cn

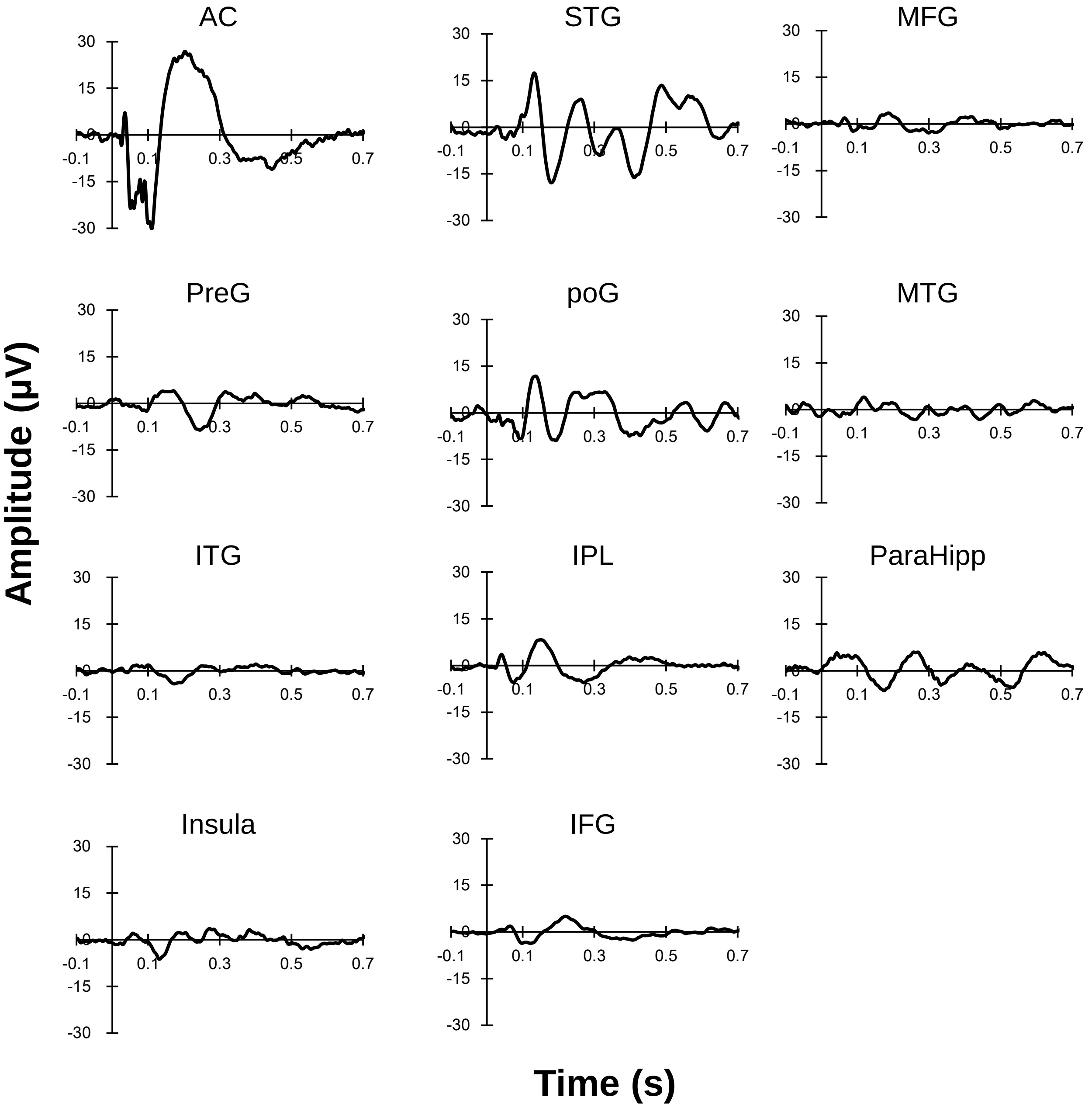

Supplementary Figure S1. Examples of raw waveforms of noise-burst-evoked potentials in representative brain areas. *AC:* auditory cortex; *PreG:* precentral gyrus; *STG:* superior temporal gyrus; *poG:* postcentral gyrus; *IPL:* inferior parietal lobule; *IFG:* inferior frontal gyrus; *MFG:* middle frontal gyrus; *MTG:* middle temporal gyrus; *ParaHipp:* para-hippocampus; *ITG:* inferior temporal gyrus.

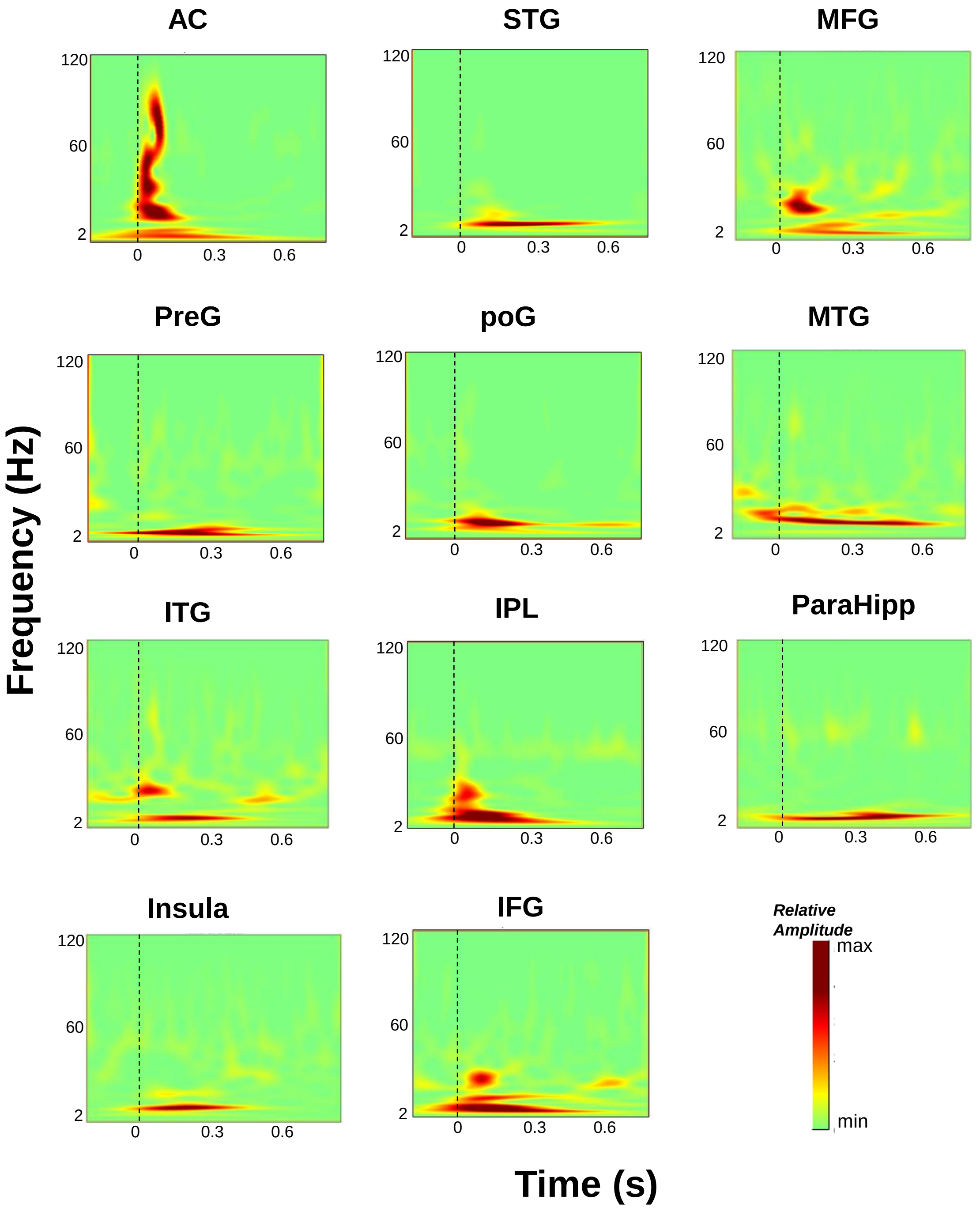

Supplementary Figure S2. Examples of time-frequency analyses of noise-burst-evoked potentials in representative brain areas. *AC:* auditory cortex; *PreG:* precentral gyrus; *STG:* superior temporal gyrus; *poG:* postcentral gyrus; *IPL:* inferior parietal lobule; *IFG:* inferior frontal gyrus; *MFG:* middle frontal gyrus; *MTG:* middle temporal gyrus; *ParaHipp:* para-hippocampus; *ITG:* inferior temporal gyrus.

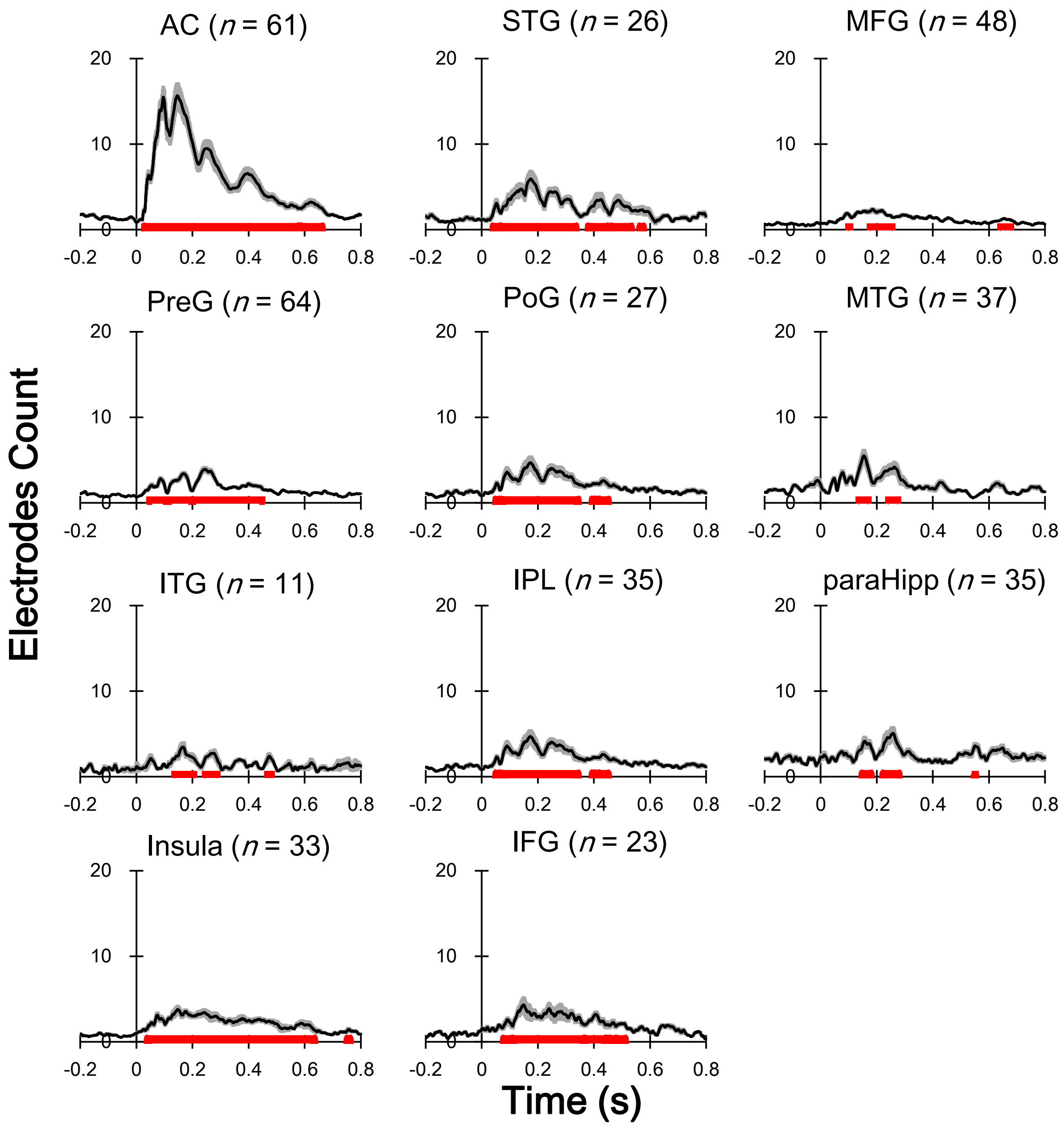

Supplementary Figure S3. Averaged waveform (absolute value) of each significantly activated brain area. Student *t*-tests against baseline (-50 to 0 ms before sound onset) with Bonferroni correction were conducted to decide the significance. *Black lines:* mean value; *Gray areas:* standard error; *Red bars:* significant temporal area. *AC:* auditory cortex; *PreG:* precentral gyrus; *STG:* superior temporal gyrus; *poG:* postcentral gyrus; *IPL:* inferior parietal lobule; *IFG:* inferior frontal gyrus; *MFG:* middle frontal gyrus; *MTG:* middle temporal gyrus; *ParaHipp:* para-hippocampus; *ITG:* inferior temporal gyrus.
